# Nanoantidotes: A Detoxification System More Applicable to Clinical Practice

**DOI:** 10.34133/bmef.0020

**Published:** 2023-05-18

**Authors:** Jiazhen Yang, Jianxun Ding

**Affiliations:** ^1^ Key Laboratory of Polymer Ecomaterials, Changchun Institute of Applied Chemistry, Chinese Academy of Sciences, 5625 Renmin Street, Changchun 130022, P. R. China.; ^2^ School of Applied Chemistry and Engineering, University of Science and Technology of China, 96 Jinzhai Road, Hefei 230026, P. R. China.

Since 2010, there has been a much greater awareness of the problem of intoxication, which has become the most common form of accidental and intentional death. In 2020, more than 90,000 people died of intoxication in the United States, which surpassed deaths due to motor vehicle accidents and firearms use to become the leading external cause of mortality. For clinical context, the causes of intoxication could be divided into 3 categories: (a) accidental ingestion of unknown xenobiotics, (b) excessive ingestion of bioactive molecules, and (c) inevitable toxicity arising from highly toxic medical drugs. However, the limited role of nonspecific extracorporeal techniques and the finite number of well-established antidotes are incapable of dealing with the complex emergencies arising from various forms of intoxication. Considering the modular properties of biocompatibility, binding affinity, biodistribution profile, and circulation time required to constitute a detoxification system, nanoantidotes would form a superior detoxification system more applicable to clinical practice [1].

Some patients do not know what specific types of xenobiotics they have accidentally ingested as a reason for intoxication. This uncertainty prevents clinicians from providing reliable detoxification programs in first-case scenarios, severely affecting patient prognoses and even threatening their lives. Broad-spectrum systems, such as intravenous lipid emulsions, have been used clinically to extract xenobiotics from tissues and enhance metabolism. However, increases in brain and heart toxicities have been occasionally reported due to reversible entrapment between lipid droplets and xenobiotics. Therefore, apart from the hydrophobicity of toxic agents, their acid–base properties would also be considered necessary in strengthening the interaction in the design of broad-spectrum nanoantidotes. For example, protonation changes the hydrophilicity of xenobiotics, and this avoids back diffusion and prevents secondary injury if the transition occurs inside a transmembrane pH-gradient trap [2]. Moreover, it is possible to use nanoantidotes to directly adsorb xenobiotics, which requires an antifouling layer, such as poly(ethylene glycol), to prevent nonspecific adsorption of the protein corona. These broad-spectrum nanoantidotes could be used for all patients in the early stages of intoxication, buying time for diagnosis and therapy.

Bioactive molecules are potential drugs and nutrients, but they also prove to be potent toxins. This blurring of pharmacological boundaries depends on their dosage. For situations of excessive exposure to definite poisons, conventional antidotes, such as small molecules and enzymes, are disappointing in terms of their biocompatibility, pharmacokinetics, and stability, which has often led to poor clinical outcomes. The nanoscale delivery systems have been the first choice for dealing with such problems. Compared with the drug-loaded detoxification systems, nanoantidotes without drugs may be more appropriate for clinical practice. By covalently modifying the different binding groups of toxic agents on a standard nanoplatform, a vast library of nanoantidotes could be developed to screen out “chemical antibodies” for xenobiotics. This drug-free detoxification strategy not only avoids the problems of large-scale production due to the complexity of components used in the drug delivery systems but also reduces the potential risks arising from issues with biocompatibility, immunogenicity, and other factors. At the same time, artificial intelligence could be used to automate the design process, simplifying the establishment of standardized preparation schemes. Moreover, biomimetic nanoantidotes that mimic natural toxin-binding sites or toxin-elimination mechanisms have also shown promising effects in proof-of-concept experiments [3]. The current limitations on the systemic detoxification of specific poisons in clinic are partly due to a lack of appropriate treatment measures. The development of drug-free nanoantidotes is bound to provide new guidance and directions for clinical practice.

Another tricky problem for clinicians is the inevitable toxicity arising from treatment with highly toxic drugs. For example, most patients cannot withstand chemotherapy due to their low tolerance to chemotherapeutic agents, leading to interruptions in treatment courses. Small-molecule antidotes reduce the systemic damage done by drugs, but the lower toxicity always comes at the expense of curative efficacy. The rise of nanomedicines has offered hope of overcoming this predicament. However, nanomedicines have negligible advantages over standalone drugs in their clinical treatment effects, or even worse. Nanoantidotes might be able to actively or passively select organs that need detoxification through molecular recognition or the regulation of size and morphology [4]. It is worth noting that the detoxification programs generally follow a 2-step administrative principle, that is, nanoantidotes need to be administrated before or at the same time as the standalone drug for pre-enrichment at the specific sites to drive superior efficacy (Figure). In this way, a higher drug dose could be safely administered to enhance its effectiveness on the lesion regions. As a result, this advanced capability of nanoantidotes is expected to optimize the therapeutic window for a range of drugs, and partial drugs aborted due to their high toxicity would be returned to clinic.

**Figure.**
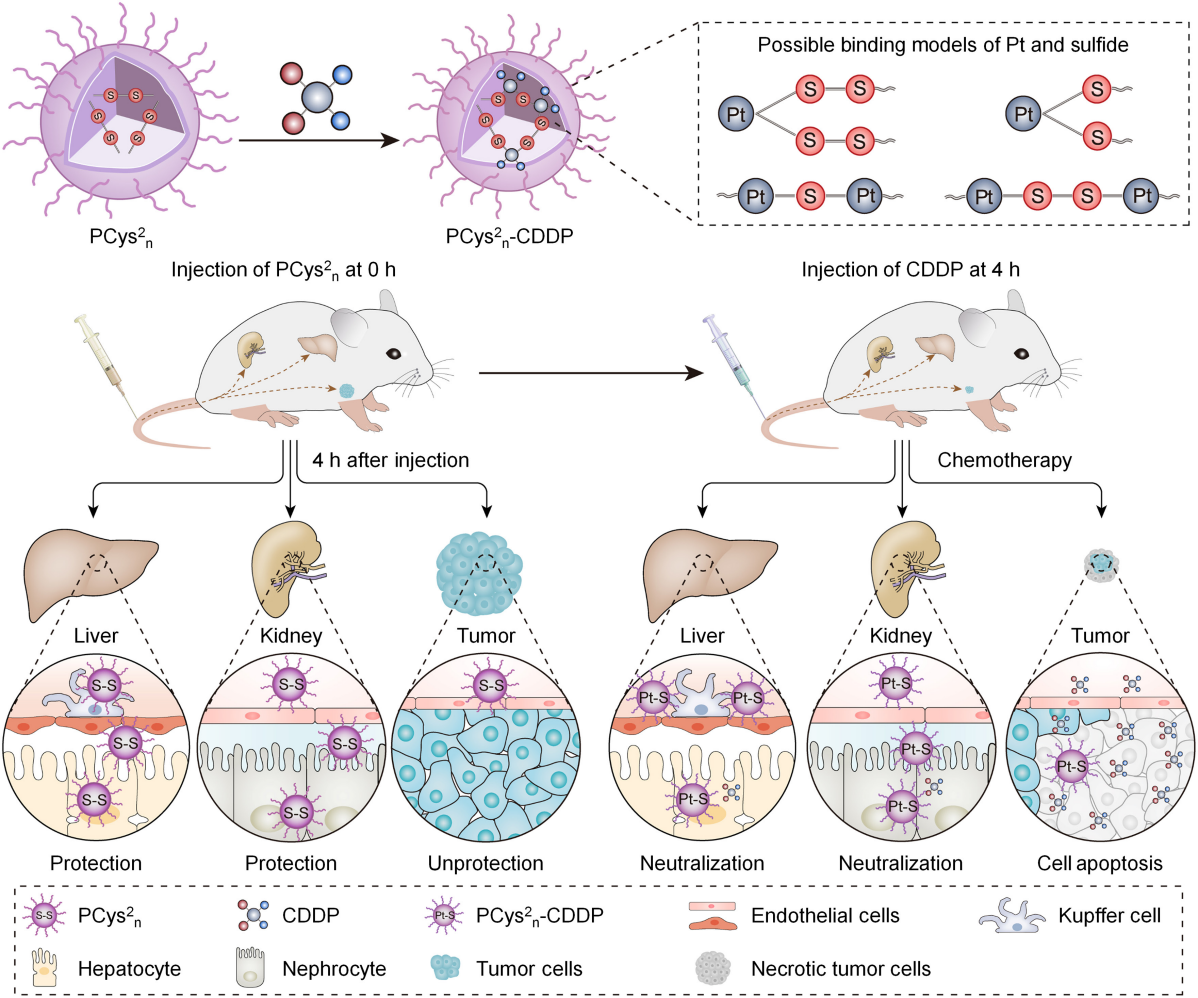
A polypeptide (PCys^2^_n_)-based detoxification system for cisplatin (CDDP) that follows the 2-step administration principle. Reprinted with permission from [4]; copyright (2022) John Wiley & Sons.

These intravenous approaches must not represent all the uses of nanoantidotes. Microneedles and hydrogels allow precisely targeted local delivery of nanoantidotes to intoxicated organs, and a mesh containing the immobilized nanoantidotes provides more options for extracorporeal blood purification. Meanwhile, preventing the poisoning of workers in some toxic work environments might be another application scenario of nanoantidotes. Combining a universal detoxification platform and efficient detoxification groups is the basis for the clinical utilization of nanoantidotes. If the modular information has been collected on an integrated barcode chip, it might be possible to prepare an all-in-one theranostic platform based on blood detection. Concomitant with that, clinically relevant rational models are essential to simulate realistic situations of chemical or biological intoxication. As for researchers, it is critical for them to understand the pharmacokinetics of nanoantidotes and toxic agents fully. Under the above guidelines, nanoantidotes would gradually become among the best choices for treating intoxication.

## References

[1] Manek E, Petroianu GA. Brain delivery of antidotes by polymeric nanoparticles. J Appl Toxicol. 2021;41(1):20–32. 32666582 10.1002/jat.4029

[2] Forster V, Signorell RD, Roveri M, Leroux JC. Liposome-supported peritoneal dialysis for detoxification of drugs and endogenous metabolites. Sci Transl Med. 2014;6(258):258ra141. 10.1126/scitranslmed.3009135 25320233

[3] Wang D, Ai X, Duan Y, Xian N, Fang RH, Gao W, Zhang L. Neuronal cellular nanosponges for effective detoxification of neurotoxins. ACS Nano. 2022;16(11):19145–19154. 36354967 10.1021/acsnano.2c08319

[4] Yang J, Su T, Zou H, Yang G, Ding J, Chen X. Spatiotemporally targeted polypeptide nanoantidotes improve chemotherapy tolerance of cisplatin. Angew Chem Int Ed. 2022;61(47): e202211136. 10.1002/anie.202211136 36069260

